# Asymmetric growth of root epidermal cells is related to the differentiation of root hair cells in *Hordeum vulgare* (L.)

**DOI:** 10.1093/jxb/ert300

**Published:** 2013-09-16

**Authors:** Marek Marzec, Michael Melzer, Iwona Szarejko

**Affiliations:** ^1^Department of Genetics, Faculty of Biology and Environmental Protection, University of Silesia, Katowice 40-032, Poland; ^2^Department of Physiology and Cell Biology, Leibniz Institute of Plant Genetics and Crop Plant Research (IPK), Gatersleben D-06466, Germany

**Keywords:** Atrichoblast, cell pattern, differentiation, epidermis, *Hordeum vulgare (barley)*, root hair, trichoblast.

## Abstract

The root epidermis of most vascular plants harbours two cell types, namely trichoblasts (capable of producing a root hair) and atrichoblasts. Here, *in vivo* analysis, confocal laser-scanning microscopy, transmission electron microscopy, histological analysis, and three-dimensional reconstruction were used to characterize the cell types present in the barley root epidermis and their distribution in the tissue. Both trichoblasts and atrichoblasts were present in the wild-type cultivars and could be distinguished from one another at an early stage. Trichoblast/atrichoblast differentiation depended on asymmetric cell expansion after a period of symmetrical cell division. After asymmetric growth, only the shorter epidermal cells could produce root hairs, whereas the longer cells became atrichoblasts. Moreover, the root epidermis did not develop root hairs at all if the epidermal cells did not differentiate into two asymmetric cell types. The root hairless phenotype of *bald root barley* (*brb*) and *root hairless 1.b* (*rhl1.b*) mutants was caused by a mutation in a gene related to the asymmetric expansion of the root epidermal cells. Additionally, the results showed that the mechanism of trichoblast/atrichoblast differentiation is not evolutionally conserved across the subfamilies of the Poaceae; in the Pooideae subfamily, both asymmetric division and asymmetric cell expansion have been observed.

## Introduction

The root epidermal cells of certain plants are capable of developing root hairs, structures that serve to increase root surface area (Cormack, 1935), and, in so doing, influence plant growth, development, and adaptation to various abiotic stresses (Parker *et al.*, 2000). The source of the root hairs is referred to as a trichoblast cell, while epidermal cells unable to generate root hairs are atrichoblasts (Libault *et al.*, 2010). In some species, any root epidermis cell can act as a trichoblast, while in others the epidermis comprises two types of cell, of which only the shorter type is capable of producing a root hair; finally, in some species, files of trichoblasts and atrichoblasts are produced (Dolan, 1996). The first of these patterns applies in most mono- and dicotyledonous species, as well as in ferns (Cormack, 1935; Dolan and Costa, 2001); the second is largely restricted to the most ancient of land plants, such as *Lycopsida* and *Sphenopsida*, the Pooideae, and a few dicotyledonous species, most prominently within the Nymphaeceae family (Bibikova and Gilroy, 2002); the final pattern has been documented in some Brassicaceae and related family species (Dolan and Costa, 2001; Pemberton *et al.*, 2001). No diagnostic morphological marker has been elaborated to detect the first type of situation (except possibly for the presence/absence of hair tubes) (Pemberton *et al.*, 2001), whereas, in the other two, trichoblasts tend to be foreshortened, their cytoplasm denser, and their level of vacuolation low (Dolan *et al.*, 1993, 1994); these features allow them to be recognized even in the meristematic zone (Dolan *et al.*, 1993). The presence of wax or other crystalline deposits on the cell surface is also diagnostic of trichoblasts (Dolan *et al.*, 1994).

The differentiation of root epidermal cells in *Arabidopsis thaliana* has provided a detailed description of the histology of root hair development (Foreman and Dolan, 2001; Bibikova and Gilroy, 2002); moreover, the genetics of root hair development has been well defined through the use of mutants (Bruex *et al.*, 2012). At least 130 genes are now known to be involved in the overall process of root hair development (Kwasniewski *et al.*, 2013) and new genes are still being identified (An *et al.*, 2012; Yoo *et al.*, 2012). A complete picture of the genes responsible for the early determination of the fate of epidermal cells in *A. thaliana* has been provided by Schiefelbein *et al.* (2009) and Song *et al.* (2011). The major genes underlying the establishment of the file-like pattern of trichoblasts and atrichoblasts are *GLABRA3* (*GL3*; Bernhardt *et al.*, 2003), *ENHANCER OF GLABRA3* (*EGL3*; Masucci *et al.*, 1996), *TRANSPARENT OF GLABRA* (*TTG*; Galway *et al.*, 1994), and *WEREWOLF* (*WER*; Lee and Schiefelbein, 1999). Loss of function of any of these four genes produces profuse hairiness. In contrast, mutations in *CAPRICE* (*CPC*; Wada *et al.*, 1997), *TRIPTYCHON* (*TRY*; Schellmann *et al.*, 2002), or *ENHANCER OF TRY AND CPC1* (*ETC1*; Kirik *et al.*, 2004) abolish root hair formation. Knowledge of the genetic basis of root hair morphogenesis outside of *A. thaliana*, and especially in monocotyledonous species, is very limited. A few root hair mutants have been described in monocotyledonous species (Wen and Schnable, 1994; Engvild and Rasmussen, 2004; Rebouillat *et al.*, 2009). Cereal genes known to influence root hair tube elongation include the *Oryza sativa* genes *OsCSLD1*, which encodes a cellulose synthase (Kim *et al.*, 2007), *OsAPY1*, which encodes an apyrase protein (Yuo *et al.*, 2009), *OsFH1*, which encodes a rice formin homology (Huang *et al.*, 2013a), and *OsSNDP1*, which encodes a Sec14-nodulin domain-containing protein (Huang *et al.*, 2013b), along with the maize genes *RTH1*, which encodes a protein involved in polar exocytosis (Wen *et al.*, 2005), and *RTH3*, which encodes a COBRA-like protein (Hochholdinger *et al.*, 2008).

The literature indicates that barley is a species in which any epidermal cell can potentially form a root hair (Clowes, 2000). Gene identification in barley is facilitated by a collection of root hair mutants in barley assembled by Szarejko *et al.* (2005); these stocks, classified according to their effect on phenotype, fall into four groups: those producing no root hairs (*rhl*), those producing only primordia (*rhp*), those producing foreshortened root hairs (*rhs*), and those that develop an irregular distribution of root hairs (*rhi*; B. Chmielewska *et al.*, unpublished). So far, the only barley gene potentially involved in root hair growth and development has been the gene *HvEXPB*, which encodes an expansin protein (Kwasniewski and Szarejko, 2006). Knowledge about the first stages of root hair differentiation, such as the establishment of trichoblasts and atrichoblasts, or the initiation of root hair formation, is especially limited. Histological analyses of root hair morphogenesis and comparative studies of root hair mutants and their parent varieties will facilitate the understanding of these processes. Additionally, the results obtained during histological research will enable new candidate genes that are involved in root hair development to be typed, as in the case of *A. thaliana*.

The present report describes an analysis of the pattern of root epidermal cells in certain barley root hair mutants in an attempt to characterize the process of trichoblast/atrichoblast formation. We showed that trichoblasts and atrichoblasts can be distinguished from one another at a very early stage of differentiation of the root epidermis, during the phase of asymmetric cell growth. Neither of the two epidermal cell types was present in root hairless mutants, indicating that asymmetric growth is essential for trichoblast differentiation in barley.

## Material and methods

### Plant material and growing conditions

The barley mutants *rhl1.b*, *rhp1.a*, *rhs.3a*, and *rhi1.a* were isolated after chemical mutagenesis with methylnitrosourea and sodium azide in the Department of Genetics, University of Silesia (Szarejko *et al.*, 2005). The *brb* mutant was obtained from Dr T. Gahoonia (Royal Veterinary and Agricultural University, Denmark) and the *rhi2.d* and *rhi3.a* mutants from Dr B. Foster (James Hutton Institute, Scotland, UK; currently IAEA, Vienna). Four different background cultivars were used to obtain the various mutants, which, with the exception of *brb* and *rhl1.b*, are all known to be non-allelic (Table 1). Grains were surface sterilized by immersion in 20% household bleach and germinated in glass tubes sealed with Parafilm, following the method of Szarejko *et al.* (2005). Seedlings were raised under a 16h photoperiod at 20 °C and provided with 180 μE m^–2^ s^–1^ of light. Tissue was sampled when the seedlings were 5 d old.

**Table 1.** Barley root hair mutants and the parent cultivars used

Phenotype	Gene symbol	Mutant name	Parent cultivar
Totally root hairless mutants; no cell forms the root hair	*rhl1.b*	*root hairless 1.b*	‘Karat’
	*brb*	*bald root barley*	‘Pallas’
The differentiation of root hairs is inhibited at the primordium stage; trichoblasts produce only bulges that were unable to enter tip growth	*rhp1.a*	*root hair primordia 1.a*	‘Dema’
Root hairs shorter than in parent variety by 60%.	*rhs3.a*	*root hair short 3.a*	‘Karat’
In the mature root hair zone, sparsely located root hairs of different lengths are present. Irregular density of root hairs along the roots	*rhi1.a*	*root hair irregular 1.a*	‘Dema’
	*rhi2.d*	*root hair irregular 2.d*	‘Optic’
	*rhi3.a*	*root hair irregular 1.a*	‘Optic’

### Light and transmission electron microscopy (TEM)

For histological and ultrastuctural examinations, combined conventional and microwave-assisted fixation, substitution, and embedding of 2mm root segments were performed using a PELCO BioWave 34700-230 (TedPella, Redding, CA, USA), according to the procedure described by Thiel *et al.* (2012). For histological examination, semi-thin sections (~2 μm thick) were cut from the embedded samples, mounted on slides, and stained for 2min with 1% (w/v) methylene blue/1% (w/v) Azur II in 1% (w/v) aqueous borax at 60 °C prior to light microscopic examination with a Zeiss Axiovert 135 microscope. For electron microscopic analysis with a Tecnai Sphera G2 (FEI Company, Eindhoven, The Netherlands) transmission electron microscope at 120kV, ultrathin sections of ~70nm thickness were cut with a diamond knife and contrasted with a saturated methanolic solution of uranyl acetate and lead citrate before examination.

### Fluorescence and confocal laser-scanning microscopy (CLSM)

Root samples (a minimum of seven roots per entry, allowing analysis of >1050 epidermal cells) were treated with 0.2mg ml^–1^ of fluorescein diacetate (FDA; Sigma-Aldrich) in de-mineralized water in the dark for 10min, and then washed in 200ml of de-mineralized water, placed on a glass slide, and covered with a cover slip. Emission was detected with an argon 488nm laser line equipped with a 505–550nm band-pass filter. Autofluorescence was detected with a 364nm UV laser line equipped with a 375nm band-pass filter.

Nuclei in the root epidermal cells were visualized by fixing the roots in 2% (v/v) formaldehyde, 2% (v/v) glutaraldehyde in 50mM cacodylate buffer (pH 7.2), washing three times in distilled water, staining in 1mg l^–1^ of 4′,6-diamidno-2-phenylindole (DAPI) for 15min, and washing in 200ml of de-mineralized water; the stained roots were mounted on a glass slide and covered with a cover slip. Nuclei were detected using a 364nm laser line equipped with a 385 long-pass filter, while the fluorescence of the cytoplasm was captured by an argon 488nm laser equipped with 560–615nm band-pass filter. The length of the daughter cells was measured in both the meristematic zone and after the shootward-last cell division. For this analysis, 61 roots from 30 plants of variety ‘Karat’ were fixed and stained with DAPI. The length of 272 daughter cells was measured in the meristematic zone and the length of 336 daughter cells after the shootward-last cell division was measured.

The epidermis layer in the mature root hair zone of cv. ‘Karat’ and of the *rhl1.b* mutant seedlings was observed by epifluorescence microscopy, using a Mercury BX-FLA fluorescence illuminator and a 530–550nm band-pass filter. At least 500 epidermal cells from 10 roots were measure for cv. ‘Karat’ and the *rhl1.b* mutant.

### Three-dimensional (3D) cell reconstructions

The optical sections obtained by CLSM were processed using ZEN 2009 Light Edition software (Carl Zeiss MicroImaging), based on standard settings. The Fiji (http://www.fiji.sc) open-source image processing package was used to reconstruct 3D images from the histological serial sections. Images were aligned to obtain the stack and it was them imported to the TrackEM plug-in, in which individual cells were marked manually. The 3D reconstruction was based on these manually selected cells.

### Statistical analysis

A χ^2^ test (*P*<0.05) was used to test whether the proportions of the three epidermal cell arrangement types was non-random and to compare the distribution of cell arrangements between the various mutants and their corresponding wild-type cultivar. Mean values of cell size and/or volume in various zones of the root were compared using Student’s *t-*test (*P*<0.05).

## Results

### Wild-type barley forms two types of epidermal cell in the root elongation zone

**Fig. 1.**
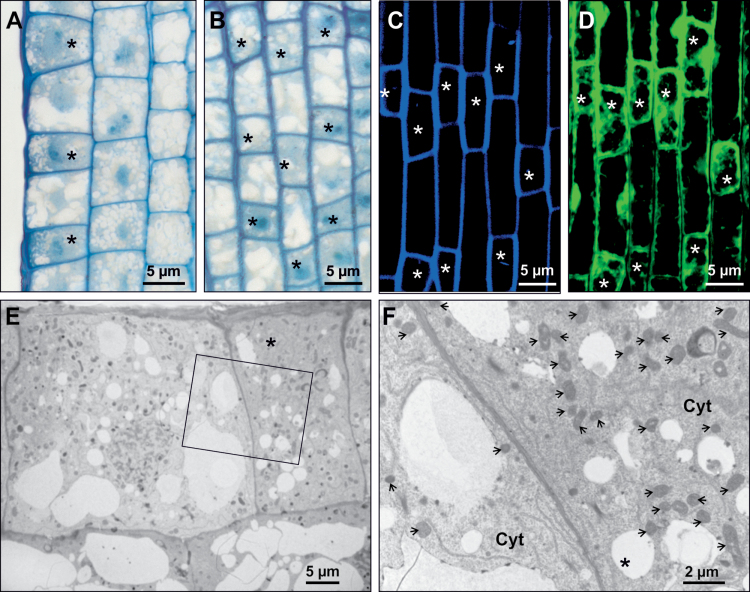
Histological analysis of epidermal cells in the elongation zone of barley root. (A, B) Light microscopy images of radial sections of a cv. ‘Karat’ root (A) and tangential sections through the epidermis of a cv. ‘Karat’ root (B). (C) Autofluorescence of the cell wall analysed by CLSM. (D) Fluorescence of the cytoplasm following FDA staining. (E, F) Ultrastructural analysis reveals variation between shorter and longer cells with respect to their cytoplasm density (E) and the number of mitochondria present (F; inset from E). Asterisks indicate trichoblasts and arrows indicate mitochondria. CW, cell wall; Cyt, cytoplasm.

**Fig. 2.**
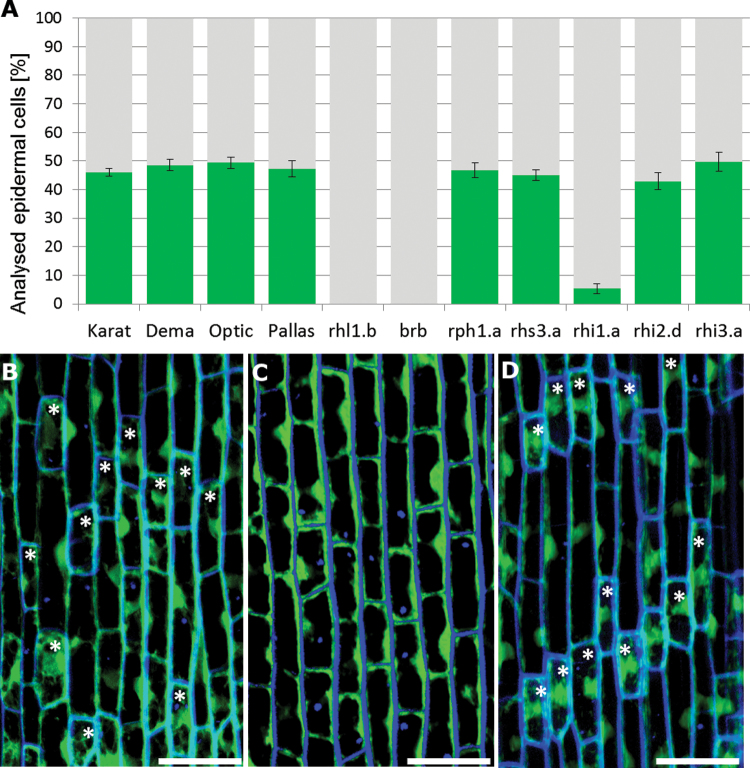
Shorter and longer epidermal cells in the elongation zone of barley roots. (A) Proportions of the two types of cell present in the root epidermis. Green represents shorter cells and grey represents longer cells. (B–D) Optical sections through the epidermal layer of cv. ‘Dema’ (B), mutant *rhl1.b* (C), and mutant *rhi2.d* (D) after staining the cytoplasm with an FDA solution. Asterisks indicate shorter cells (dense cytoplasm). Bars, 50 μm.

Once cell division ceases in the root meristem, the epidermal cells begin to differentiate into either root hair or non-root hair cells. Histological sections of the elongation zone of wild-type roots revealed two classes of epidermal cells, one being somewhat shorter than the other. Both types were represented in all cell files of the root epidermis. As the cytoplasm of the shorter cells was denser than that of longer cells (Fig. 1A, B), it was inferred that the former were trichoblasts and the latter atrichoblasts. The differences between the two classes of epidermal cells were confirmed by CLSM analysis, as the cell walls of the shorter cells autofluoresced more strongly (Fig. 1C) than those of the longer cells, and the signal emitted following FDA staining confirmed that the cytoplasm of the former was more dense (Fig. 1D). TEM analysis showed that shorter cells harboured more mitochondria than longer cells (Fig. 1E, F), an observation that supported the notion that shorter cells were trichoblasts, as root hair production requires a certain input of energy. No obvious differences with respect to either cell-wall thickness or electron density were able to explain the distinct autofluorescence profiles. A count of the two cell types present in the four wild-type cultivar roots was based on a sample of at least 1245 epidermal cells arranged in 38 files in seven independent roots, observed prior to root hair initiation. Shorter cells comprised 46.0±1.4% of the cell population in cv. ‘Karat’, 48.6±2.0% in cv. ‘Dema’, 49.4±1.9% in cv. ‘Optic’, and 47.3±2.8% in cv. ‘Pallas’ (Fig. 2A and Supplementary Table S1 at *JXB* online).

### Types of epidermal cell present in the root elongation zone of the mutants

A population of at least 1050 epidermal cells (sampled from seven independent roots) for each mutant was observed prior to root hair initiation. In the allelic root hairless mutants *rhl1.b* and *brb*, the epidermal cells were homogeneous with respect to both their length and cytoplasm density (Fig. 2B–D). Shorter cells could, however, be recognized in the *rhi1.a*, *rhi2.d*, *rhi3.a*, *rhp1.a*, and *rhs3.a* mutants, which produced root hairs (Fig. 2A). In the *rhi1.a* mutant root, only 5.3% of the cells were classified as shorter cells, but in the other mutants, the proportion of both cell types was similar to that of the wild types (Supplementary Table S1).

### Cell pattern of epidermal cells in the root elongation zone

**Fig. 3.**
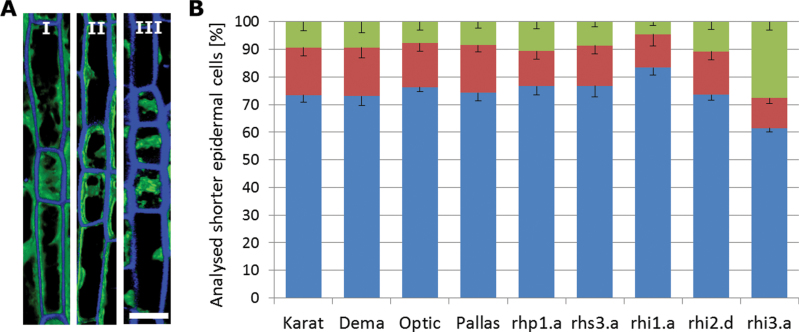
Arrangement of cells in the root epidermis. (A) In pattern I, one shorter cell is flanked by longer cells. In pattern II, a pair of shorter cells is flanked by longer cells. In pattern III, a set of three shorter cells is flanked by longer cells. Bar, 20 μm. (B) Percentage of shorter cells present in pattern I (blue), II (red), and III (green).

Three distinct patterns of epidermal cells were noted in the wild-type cultivars: in pattern I, each shorter cell was flanked on each side by a longer cell and in pattern II, a pair of shorter cells was flanked on each side by a longer cell, while in pattern III, a set of three shorter cells was flanked on each side by a longer cell (Fig. 3A). All three patterns were represented in genotypes able to produce root hairs. The most common arrangement was pattern I, which accounted for, respectively, 73.4, 73.0, 76.3, and 74.2% of the shorter cells present in the root of cvs ‘Karat’, ‘Dema’, ‘Optic’, and ‘Pallas’. Pattern III was the least frequent (ranging from 7.8% of shorter cells in cv. ‘Optic’ to 9.5% in cv. ‘Karat’) (Fig. 3B and Supplementary Table S1). Comparison of mean values of different genotypes clearly showed that there were no statistical differences (χ^2^ test; *P*<0.05) among all parental varieties, indicating that the distribution of cell arrangement is non-random (Supplementary Table S2 at *JXB* online).

The cell arrangements in the mutants *rhp1.a* and *rhs3.a* was largely similar to that in the wild-type cultivars: in the former, 12.6% of the shorter cells appeared in the context of pattern II and 10.7% of pattern III, while in *rhs3.a*, the respective proportions were 14.6 and 8.6% (Fig. 3B and Supplementary Table S1). In neither case was there any statistical difference between the mutant and its corresponding wild-type cultivar (Supplementary Table S2). The *rhi1.a* root contained many fewer shorter cells (see above), but their distribution by pattern was not statistically different from that observed in cv. ‘Dema’ (83.3, 12.1, and 4.6% present in, respectively, patterns I, II, and III) (Supplementary Table S2). The ratio of shorter to longer cells in the *rhi2.d* mutant was similar to that present in its corresponding wild-type cultivar (cv. ‘Optic’), with 73.5% of them appearing in the pattern I conformation, 15.7% as pattern II, and 10.8% as pattern III, whereas in *rhi3.a* about 61.5% of the shorter cells appeared as pattern I and 27.7% of the shorter cells appeared as pattern III, almost 20% more than in cv. ‘Optic’ (Fig. 3B and Supplementary Table S1). Only between *rhi3.a* and cv. ‘Optic’ did the difference between the proportions of cell types present reach a statistically significant level (Supplementary Table S2).

### Root hair production requires the presence of shorter epidermal cells

**Fig. 4.**
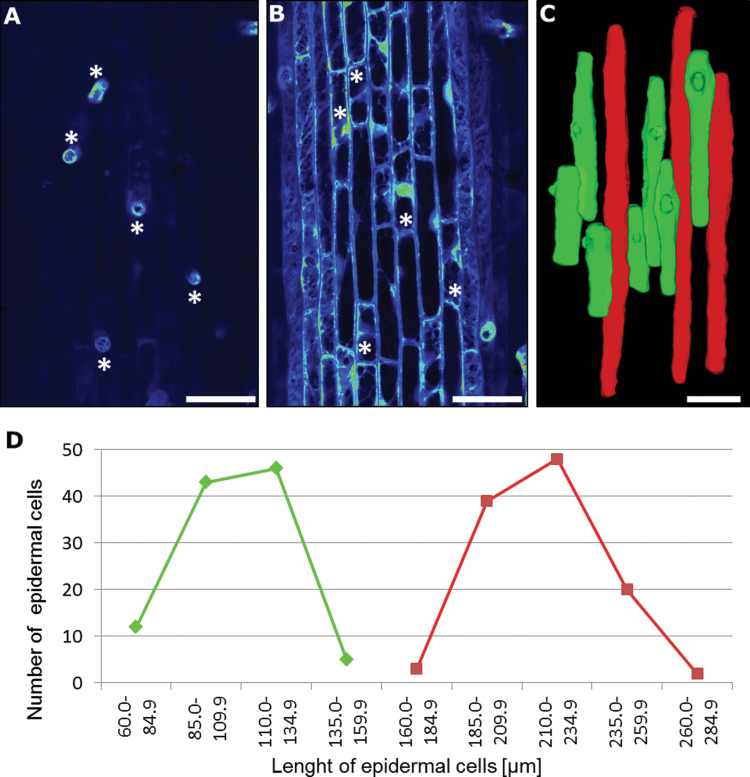
Only shorter cells are capable of producing root hairs. (A, B) CLSM visualization of the zone of root hair initiation in cv. ‘Karat’ showing the surface of the root (A) and an optical section through the epidermal layer (B). Asterisks indicate the same cell in the two images. (C) 3D reconstruction of epidermal cells within the mature zone of the root of cv. ‘Karat’. Trichoblasts are shown in green and atrichoblasts in red. (D) Length of the epidermal cells in the mature root hair zone of cv. ‘Karat’. Bars, 50 μm.

The various zones of the root were inspected to determine the location of cells capable of producing root hairs. Histological sections through the early differentiation zone (site of the earliest primordia) in all of the entries that differentiated both shorter and longer cells suggested that only shorter cells were able to develop root hairs (data not shown). The two classes of cells were clearly identifiable in the zone of rapid expansion, where it was evident that root hairs emerged exclusively from shorter cells (Fig. 4A, B). When the cv. ‘Karat’ mature root hair zone was reconstructed, it was again apparent that only shorter cells were trichoblasts (Fig. 4C). Mean epidermal cell length in the mature root hair zone, based on 106 trichoblasts and 109 atrichoblasts sampled from 10 independent cv. ‘Karat’ roots, was, respectively, 119.5±38.2 and 234.3±37.6 μm (Fig. 4D). A statistical test confirmed that these lengths differed significantly from one another. In the comparable zone of the *rhl1.b* root, where only a single type of epidermal cell was recognized, the mean epidermal cell length was 219.1±50.4 μm (Supplementary Fig. S1 at *JXB* online). Thus, it was clear that the presence of shorter cells was necessary for root hair initiation.

### Division of epidermal cells in the root meristem is symmetrical

A sample of 10 cv. ‘Karat’ roots was analysed in an attempt to establish whether the origin of the two types of epidermal cells reflected either an asymmetrical division of the mother cell, or whether they diverged in length due to an asymmetrical expansion of the daughter cells. The resulting population of 272 daughter cells was divided into one half located proximal to the root meristem (rootward cells), and the other half closer to the differentiation zone (shootward cells), following the terminology of Baskin *et al.* (2010). The mean length of the rootward cells was 5.7±0.4 μm, whereas the mean length of the shootward cells was 5.9±0.4 μm (Supplementary Fig. S2 at *JXB* online), and these lengths did not differ significantly from one another, according to Student’s *t-*test (*P*<0.05). The implication was that the divisions in the meristematic zone of root were symmetrical.

**Fig. 5.**
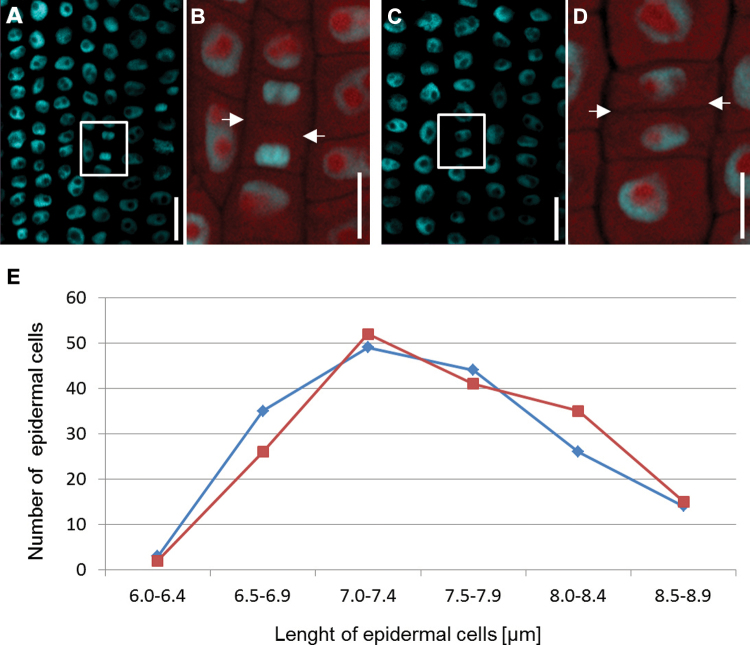
Shootward-last division of epidermal cells in a cv. ‘Karat’ root. (A) The highlighted region indicates daughter cells at late mitosis. (B) Fluorescence of the cytoplasm identifies the cell plate (indicated by arrows). (C) Compact nuclei of epidermal cells immediately post-cytokinesis. (D) Cytoplasm fluorescence, permitting an estimate of cell length (arrows indicate the cell wall separating adjacent daughter cells). Nuclei in (A)–(D) were stained with DAPI. Bars, 10 μm. (E) Lengths of the shootward (blue) and rootward (red) cells after the shootward-last cell division.

However, most important for the fate of cells is the shootward-last cell division, which is the last division in the cell file, located farthest from the root meristem. Asymmetrical division would directly indicate the type of differentiation of the daughter cells. On the other hand, in the case of asymmetrical division, the first stage of differentiation would be the asymmetrical expansion of daughter cells. A sample of 61 cv. ‘Karat’ roots was therefore fixed and stained with DAPI (Fig. 5A–D), and either the last distinguishable mitosis, showing a visible cell plate between daughter cells (Fig. 5B), or two daughter cells harbouring small, compact nuclei characteristic of a recent cell division (Fig. 5D), was identified. The autofluorescence of the cytoplasm was taken as an indicator of cell size. Based on a sample of 336 cells, the estimated length of the rootward cells was 7.56±0.58 μm, whereas that of the shootward cells was 7.52±0.59 μm; these values did not differ statistically from one another. The difference between daughter cell length was never greater than 0.83 μm (~11% of the total cell length), and no correlation could be established between cell length and the position of the cell after division (Fig. 5E).

**Fig. 6.**
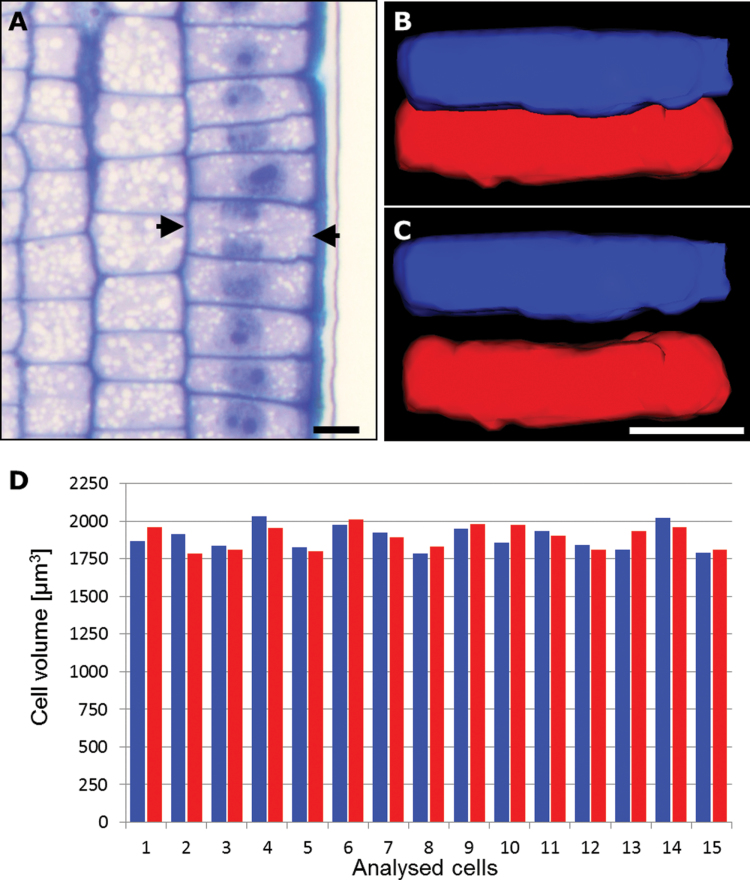
Volume of daughter cells following the shootward-last cell division. (A) Longitudinal sections through a cv. ‘Karat’ root (used for 3D reconstruction). Arrows indicate the cell plate separating adjacent daughter cells. (B, C) 3D modelling of shootward (blue) and rootward (red) cells: *in vivo* arrangement (B) and isolated cells (C). Bars, 20 μm. (D) Cell volumes of shootward (blue) and rootward (red) cells.

The CLSM data were supported by the 3D reconstruction of 30 cells selected from a sample of histological serial sections from 11 cv. ‘Karat’ roots (Fig. 6A–C). Estimates of cell volume showed that that of the rootward cells (1894.71±132.54 μm^3^) was comparable with that of the shootward cells (1786.71±112.16 μm^3^) (Fig. 6D), confirming the symmetrical nature of the shootward-last cell division. No correlation could be established between cell volume and the position of the cell after division (Fig. 6). TEM analysis of five roots confirmed the lack of any difference in cell size, density, or vacuolation, and that the structure of the cell walls of the daughter cells was homogeneous. Following a cell division, the number of mitochondria present in the two daughter cells was also comparable (Supplementary Fig. S3 at *JXB* online).

## Discussion

Although the perception has been that, in barley, trichoblasts are not readily distinguishable from atrichoblasts (Clowes, 2000), we have been able to show that these two cell types can in practice be identified quite early in the root elongation zone. The arrangement of the two cell types fits pattern II, according to the classification of Dolan (1996). The epidermal cells are arranged alternately in files, although occasionally two or three trichoblasts can develop side by side. Moreover, a similar arrangement of cells was observed in two mutants that demonstrated abnormal root hair development after root hair initiation: the *rhp1.a* mutant that produced only root hair primordia and the *rhs3.a* mutant with short root hairs. These results indicated that the cell pattern of the root epidermis in barley is not correlated with the later stages of root hair development after formation of the bulge.

Two of the root hairless mutants (*rhl1.b* and *brb*) are known to be allelic. The former was induced by methylnitrosourea mutagenesis of cv. ‘Karat’ and the latter was a spontaneous mutant in cv. ‘Pallas’. In both mutants, the differentiation of the epidermal cells into shorter and longer cells failed to occur. As only shorter cells can act as trichoblasts, the indication is that the gene disrupted in both mutants must be involved in the early stage of cell pattern formation. The remaining mutants formed root hairs, although they were compromised with respect to either their extent of development or their distribution. In *rhi1.a*, the reduction in root hair density and the irregular distribution of root hairs flowed from the drastic decrease in the number of trichoblasts formed (only ~5% of cells, compared with ~50% in the wild type). Thus, the mutated gene in this case appeared to be involved in a similar part of the root hair cell differentiation process to that which is compromised in *rhl1.b/brb* mutants. In contrast, *rhi2.d* and *rhi3.a* mutants produced a similar proportion of trichoblasts to their wild-type parental cultivar, which implies that the genes mutated in these genotypes must be involved in later stages of root hair development.

In *A. thaliana*, some of the genes determining epidermal cell fate (*GL3*, *EGL3*, and *CPC*) encode proteins that are symplasmically transported between neighbouring cells (Bernhardt *et al.*, 2005; Kurata *et al.*, 2005). Thus, a possible biochemical basis for the phenotype of the barley mutants lies in some failure in processes related to the symplasmic isolation of epidermal cells, thereby inhibiting their asymmetrical expansion (Marzec *et al.*, 2013). It is well established that cell-to-cell communication through the plasmodesmata is an important component of the determination of the direction of cell differentiation (Rinne and van der Schoot, 1998; Ruan *et al.*, 2004; Kim and Zambryski, 2005). During the differentiation of *A. thaliana* root epidermal cells into trichoblasts and atrichoblasts, the extent of symplasmic communication between adjacent epidermal cells has been shown to be reduced (Duckett *et al.*, 1994). Three alternative models of root epidermal cell differentiation in *A. thaliana* have been proposed, all involving the movement of proteins through the plasmodesmata (Benítez *et al.*, 2008; Petricka and Benfey, 2008; Savage *et al.*, 2008). It is known that symplasmic isolation is necessary for certain developmental processes, including the development of the embryo sac in *Torenia fournieri* (Han *et al.*, 2000; Dresselhaus, 2006) and the establishment of the apical–basal axis and tissue differentiation in the course of embryogenesis in *A. thaliana* (Kim and Zambryski, 2005; Kim *et al.*, 2005; Stadler *et al.*, 2005). In each of these processes, either isolated cells or groups of cells initiate a developmental programme as soon as communication with their neighbouring cells via the plasmodesmata is disrupted. The hypothesis that the gene disrupted in the *rhl1.b*/*brb* mutants encodes a protein related to symplasmic communication has, however, yet to be verified experimentally.

Kim and Dolan (2011), based on their own studies in *Brachypodium distyachon* and *O. sativa* and studies in the literature (Leavitt, 1904; Row and Reeder, 1957; Clarke *et al.*, 1979), collected information on the formation of the root epidermal cell pattern in the Poaceae family. In both species analysed, two types of epidermal cell could be distinguished—a smaller root hair-bearing cell and a larger, non-root hair cell. These two cell types derived either from asymmetrical divisions (*B. distayachon*) or from the asymmetrical growth of the epidermal cells (*O. sativa*) (Kim and Dolan, 2011). The presented classification of the two types of cell differentiation is consistent in Poaceae subfamilies; for example, all of the analysed representatives showed symmetrical cell divisions in Chloridoideae (Leavitt, 1904; Row and Reeder, 1957), while in Pooideae, all of the described species represented asymmetrical cell divisions (Row and Reeder, 1957; Kim and Dolan, 2011). These results suggested that the formation of the epidermal pattern was conserved during evolution in the Poaceae family. Our results clearly showed that, in contrast to other representatives from the Pooideae subfamily, such as *B. distachyon* (Kim and Dolan, 2011), *Hordeum jubatium*, and *Triticum aestivum* (Row and Reeder, 1957), root epidermis differentiation in barley (*Hordeum vulgare*) is characterized by symmetrical divisions of the epidermal cells followed by asymmetrical cell expansion. However, only the analysis of *B. distachyon* (Kim and Dolan, 2011) was carried out using precise and modern techniques, such as CLSM, whereas other species from the Pooideae were analysed in the 1950s of the last century using only light microscopy tools (Row and Reeder, 1957). Additionally, during these analyses, just two patterns of root epidermis were described; festucoid, when only shorter cells produce root hairs, and panicoid, characterized by root epidermal cells of similar size, any of which may produce root hair (Row and Reeder, 1957). Now we know that two types of epidermal cell, trichoblasts and atrichoblasts, may develop in two different ways: after asymmetrical division of a mother cell (as in *B. distachyon*) and after asymmetrical expansion of daughter cells (as in *O. sativa*) (Kim and Dolan, 2011). The tools used in the present study, such as *in vivo* analysis, CLSM, TEM, histological sections, and 3D reconstructions, proved that the shootward-last cell division in the epidermal layer of a barley root is symmetrical. The asymmetrical expansion of daughter cells causes the differentiation of cells into trichoblasts and atrichoblasts after this symmetrical division. As mentioned earlier, the symmetrical division and asymmetrical expansion of epidermal cells is a typical way for root hair development in many species of the Poaceae family, such as *Zea mays* or *O. sativa* (Clarke *et al.*, 1979; Kim and Dolan, 2011). Our results show that the mechanism of trichoblast/atrichoblast differentiation is not evolutionally conserved across the subfamilies of the Poaceae: the representatives from the same subfamilies can develop root hairs in different ways. Nevertheless, research on additional species from the Poaceae and re-examination of early works on this field are necessary to answer the question of whether root hair development is an evolutionally conserved process in monocots.

## Supplementary data

Supplementary data are available at *JXB* online.

Supplementary Fig. S1. The length of the epidermal cells in the mature root hair zone.

Supplementary Fig. S2. Epidermal cell divisions in the meristematic zone of the cv. ‘Karat’ root.

Supplementary Fig. S3. TEM analysis reveals the similarity in size of the shootward and rootward cells following the shootward-last cell division.

Supplementary Table S1. Patterns of epidermal cell arrangement in barley root hair mutants and cultivars.

Supplementary Table S2. The distribution of the types of cell arrangement in the root epidermis among cultivars and root hair mutants.

Supplementary Data

## Abbreviations:

3D: three dimensional
CLSM: confocal laser-scanning microscopy
FDA: fluorescein diacetate
TEM: transmission electron microscopy.
