# Supplementary material for: Asymmetric growth of root epidermal cells is related to the differentiation of root hair cells in Hordeum vulgare (L.)
Source: J Exp Bot. 2013 Sep 16;64(16):5145–55. doi: 10.1093/jxb/ert300 (PMC3830489; doi:10.1093/jxb/ert300)
Supplement: Supplementary Data [file supp_64_16_5145__index.html]

Asymmetric growth of root epidermal cells is related to the differentiation of root hair cells in Hordeum vulgare (L.) — Supplementary Data 

# Asymmetric growth of root epidermal cells is related to the differentiation of root hair cells in *Hordeum vulgare* (L.)

## Supplementary Data

Data files

**Files in this Data Supplement:**

- Supplementary Data - Supplementary Data
