# Supplementary material for: Asymmetric growth of root epidermal cells is related to the differentiation of root hair cells in Hordeum vulgare (L.)
Source: J Exp Bot. 2013 Sep 16;64(16):5145–55. doi: 10.1093/jxb/ert300 (PMC3830489; doi:10.1093/jxb/ert300)
Supplement: Supplementary Data [file supp_ert300_ert300_replacement_supplementary_data.pdf]

Table S1. Patterns of epidermal cell arrangement in barley root hair mutants and cultivars. See text for explanation.

**Genotype**	**The number of analyzed cells**	**Trichoblasts**			**Pattern type I**		**Pattern type II**		**Pattern type III**	
		**N.**	**%**	**SD**	**N**	**%**	**N**	**%**	**N**	**%**
Karat	1512	698	46.0	1.4	512	73.4	120	17.2	66	9.5
Dema	1652	803	48.6	2.0	586	73.0	142	17.7	75	9.3
Optic	1245	615	49.4	1.9	469	76.3	98	15.9	48	7.8
Pallas	1492	705	47.3	2.8	523	74.2	122	17.3	60	8.5
rhl1.b	1735	0	0.0	0.0	0	0.0	0	0.0	0	0.0
brb	1472	0	0.0	0.0	0	0.0	0	0.0	0	0.0
rhp1.a	1084	506	46.7	2.6	408	76.7	87	12.6	60	10.7
rhs3.a	1398	628	45.0	1.8	320	76.8	56	14.6	144	8.6
rhi1.a	1263	66	5.3	1.8	388	83.3	64	12.1	54	4.5
rhi2.d	1290	555	42.9	2.9	482	73.5	92	15.7	54	10.8
rhi3.a	1050	520	49.7	3.3	55	61.5	8	10.8	3	27.7

Table S2. The distribution of the types of cell arrangement in the root epidermis among cultivars and root hair mutants

Genotypes	Trichoblasts [%]			
	Type I	Type II	Type III	χ^2_0
**Karat / Dema**	73 / 73	17 / 18	10 / 9	**0.16**
**Karat / Optic**	73 / 76	17 / 16	10 / 8	**0.58**
**Karat / Pallas**	73 / 74	17 / 17	10 / 9	**0.11**
**Dema / Optic**	73 / 76	18 / 16	9 / 8	**0.46**
**Dema / Pallas**	73 / 74	18 / 17	9 / 9	**0.07**
**Optic / Pallas**	76 / 74	18 / 17	9 / 9	**0.24**

Genotypes	Trichoblasts [%]			
	Type I	Type II	Type III	χ^2_0
**Dema / *rhp1.a***	73 / 77	18 / 13	9 / 11	**2.05**
**Karat / *rhs3.a***	73 / 77	17 / 15	10 / 9	**0.55**
**Dema / *rhi1.a***	73 / 83	18 / 12	9 / 5	**5.15**
**Optic / *rhi2.d***	76 / 74	16 / 15	9 / 8	**1.24**
**Optic / *rhi3.a***	73 / 74	18 / 17	8 / 11	**44.17**

χ^2 test, $P > 0.05$; $\chi^2_{0.05} = 5.99$

Figure S1.

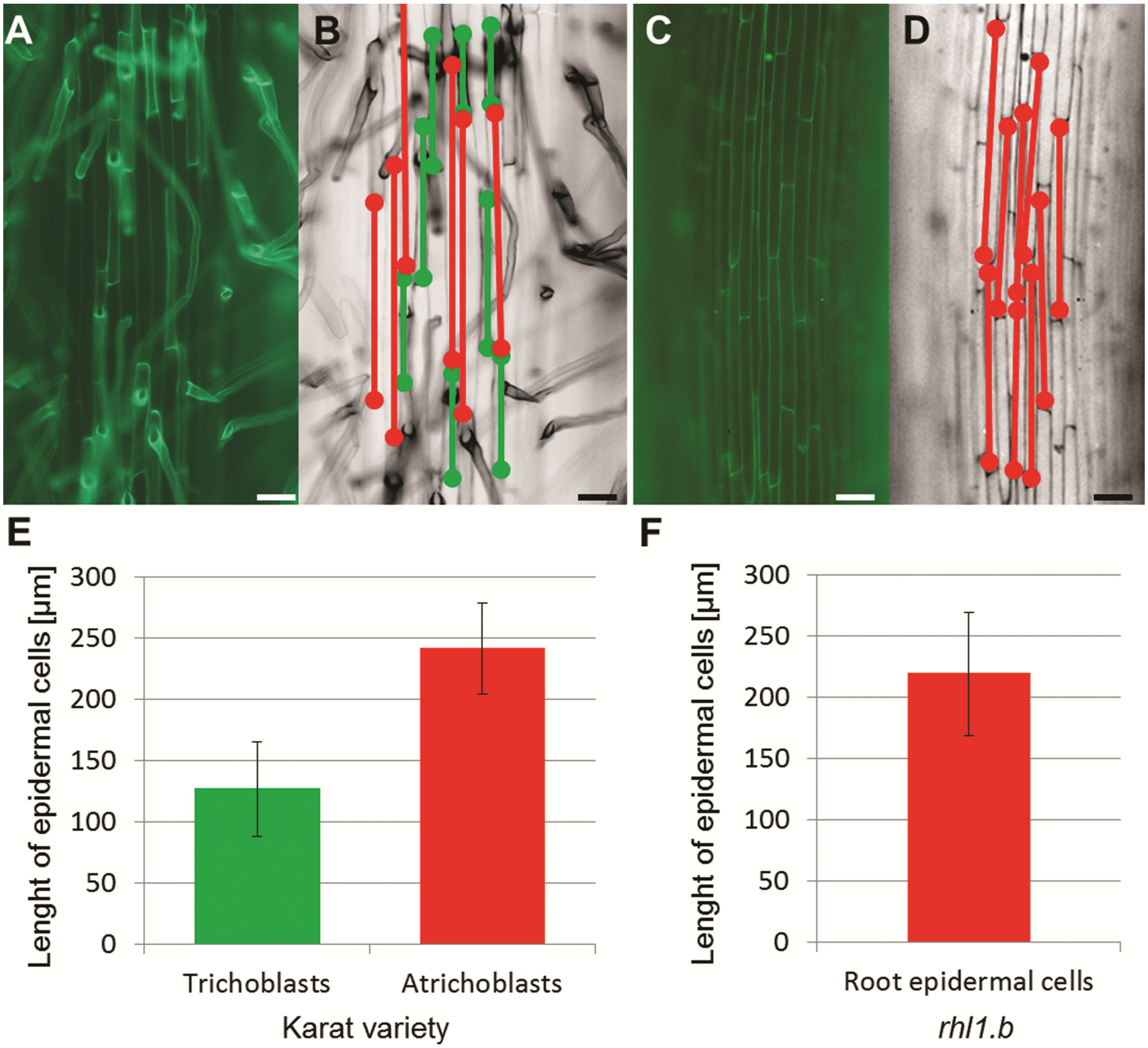

Figure S1. The length of the epidermal cells in the mature root hair zone. The epidermis layer of the cv. 'Karat' root (A) visualized under blue light, and (B) after image processing. Root epidermal cells of the *rhl1.b* mutant visualized (C) under blue light, and (D) after image processing. Cells producing root hairs are marked in green, and those not producing root hairs in red. The length of epidermal cells in the mature zone of the root of (E) cv. 'Karat', (F) *rhl1.b* mutant. Bar: 50 µm.

Figure S2.

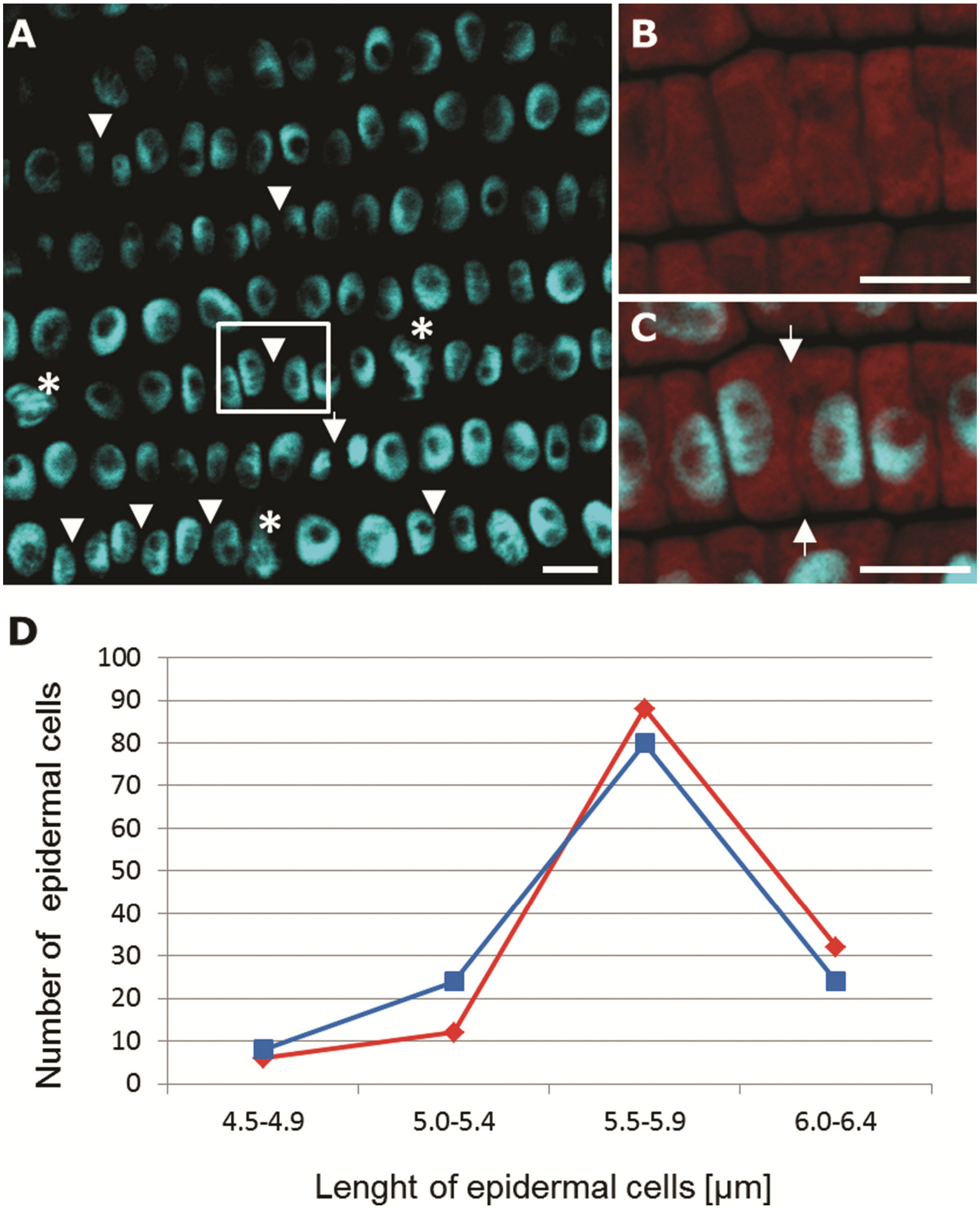

Figure S2. Epidermal cell divisions in the meristematic zone of the cv. 'Karat' root. (A) DAPI stained nuclei. Stars indicate cells experiencing mitosis, arrowheads nuclei post cell division. (B) Fluorescence of the cytoplasm after fixation, featuring the highlighted region shown in (A). (C) Merged image, combining the fluorescence signals of the nuclei and the cytoplasm. Arrows indicate the cell wall separating adjacent daughter cells. (D) The comparable length of the distal (blue) and proximal (red) cells indicates that all of the divisions were symmetrical. Bar: 10 µm.

Figure S3.

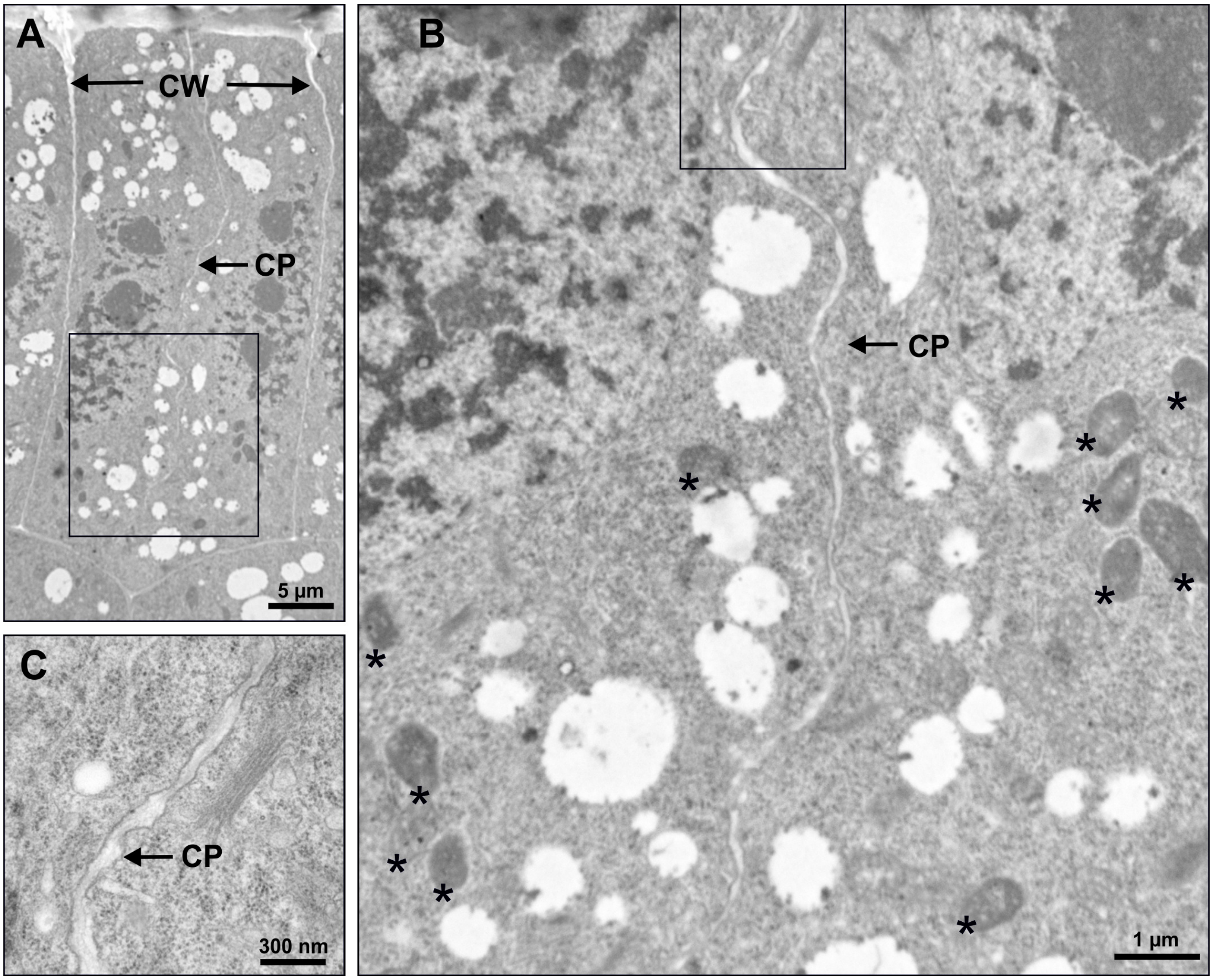

Figure S3. Figure S3. TEM analysis reveals the similarity in size of the shootward and rootward cells following the shootward-last cell division. (A) A pair of daughter cells separated by the cell plate, (B) the basal part of two daughter cells. (C) A higher magnification view of the cell plate. CW: cell wall, CP: cell plate. Stars indicate mitochondria.
